# Successful percutaneous femoral extraction of a detached tricuspid valve‐in‐valve balloon delivery system

**DOI:** 10.1002/ccr3.2307

**Published:** 2019-07-10

**Authors:** Baldeep S. Sidhu, Justin Gould, Benjamin Sieniewicz, Bradley Porter, Bernard Prendergast, Simon Redwood, Christopher A. Rinaldi

**Affiliations:** ^1^ Cardiology Department Guy’s & St Thomas' Hospitals London UK; ^2^ Division of Imaging Sciences and Biomedical Engineering King's College London London UK

**Keywords:** percutaneous lead extraction, tricuspid valve‐in‐valve, tricuspid valve‐in‐valve complication

## Abstract

We shall discuss a patient who underwent a tricuspid valve‐in‐valve implantation for a failing bioprosthetic valve replacement. The procedure was complicated by detachment of the valve deployment apparatus which was removed in its entirety, using percutaneous extraction techniques. We believe this was the first ever case to report such a complication.

## 1 INTRODUCTION

The management of severe tricuspid regurgitation (TR) in the absence of other cardiac abnormalities can be difficult, with a high risk of surgical complications.^1^ If surgery is carried out, bioprosthetic valve replacements (BVR) are preferred but, following the operation, recurrent TR may be seen within a few years. 2 Furthermore, surgical intervention for failing BVR’s is associated with a high risk of morbidity and mortality and this has led to the development of a percutaneous approach to treat such patients, using a tricuspid valve‐in‐valve (TVIV) implant.^3^ This technique involves placing a transcatheter aortic valve prosthesis within the BVR, in an off‐label indication. A multicenter registry has recently demonstrated this technique is both technically and clinically successful, with the majority of patients reporting improvement in their functional class.^4^ We shall present a case of a patient that underwent a TVIV which was complicated by device detachment and that was successfully treated using a percutaneous femoral extraction technique.

## 2 CASE REPORT

**Figure 1:**
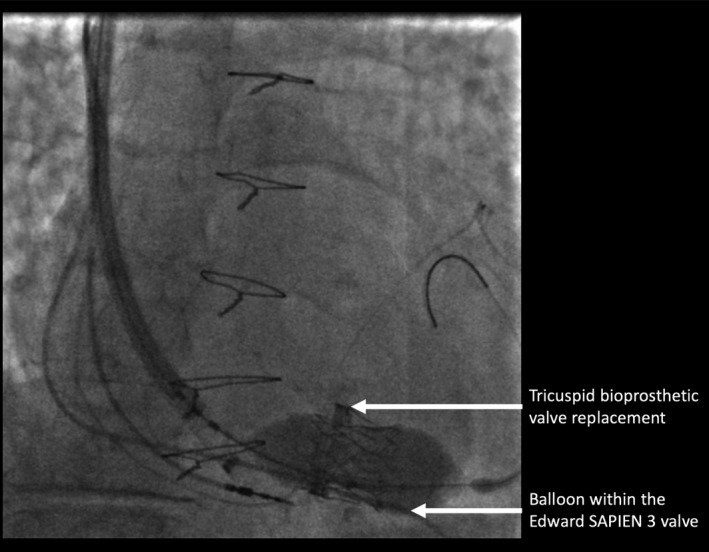
The Edward SAPIEN three heart valve was successfully deployed across the previous bioprosthetic valve replacement with an excellent result

An 87‐year‐old patient was referred for percutaneous TVIV implantation after complaining of breathlessness and peripheral edema. Three years previously, she had received a BVR for severe TR. Her functional status deteriorated, and she became increasingly breathlessness with signs of right heart failure. Echocardiography demonstrated the presence of a severe trans‐valvular leak through her BVR with preserved biventricular systolic function. Her case was discussed at a multidisciplinary meeting, it was thought that redoing tricuspid valve (TV) surgery would be too high risk, and it was decided that, instead, a TVIV implant should be performed. Initially, the procedure was performed using a femoral approach but due to difficulty achieving stable positions across the TV, the access site was changed to the right internal jugular vein (RIJV). A 26‐mm Edwards SAPIEN three heart valve was successfully deployed across the BVR and ballooned into place with an excellent result (Figure 1).

**Figure 2:**
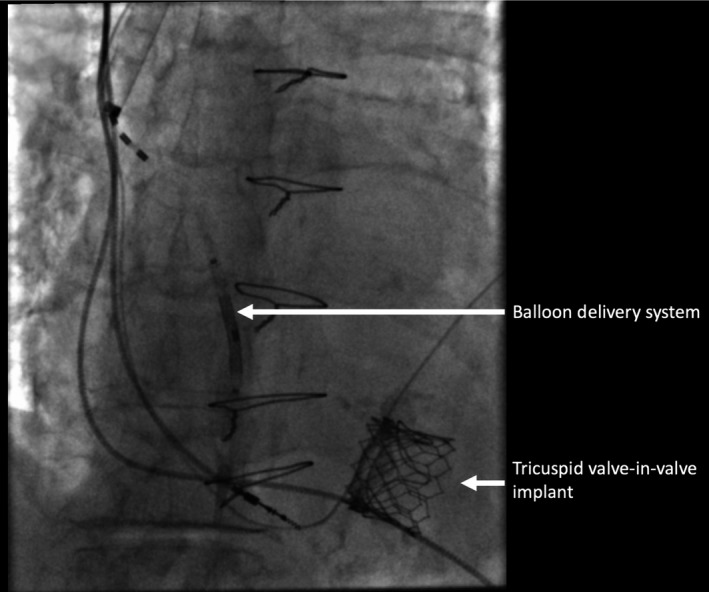
The balloon delivery system used to deploy the Edward SAPIEN three valve was entangled in the left ventricular lead, and we were unable to remove this despite multiple attempts

**Figure 3:**
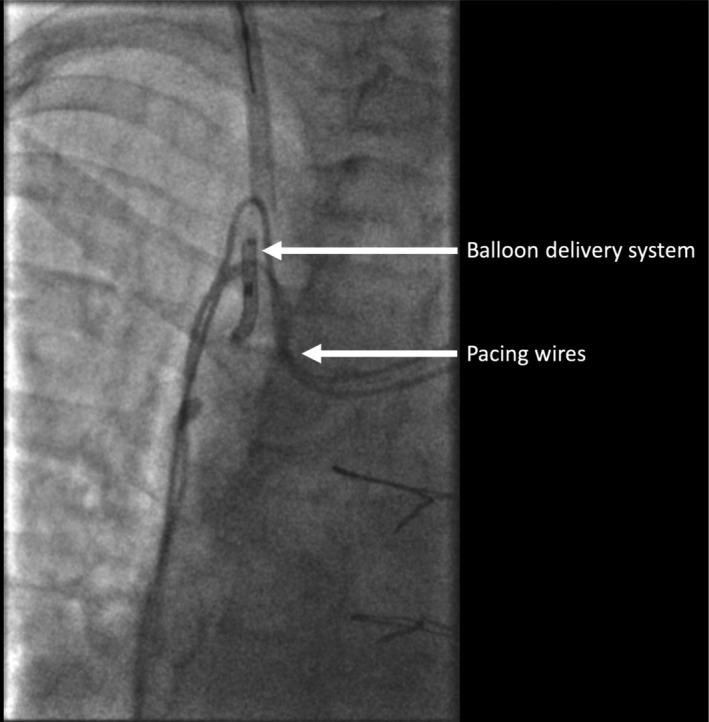
We applied continuous traction via the right internal jugular vein but were unable to remove the balloon delivery system. In this figure, the balloon delivery system can be seen trapped between the pacing leads in the superior vena cava

**Figure 4:**
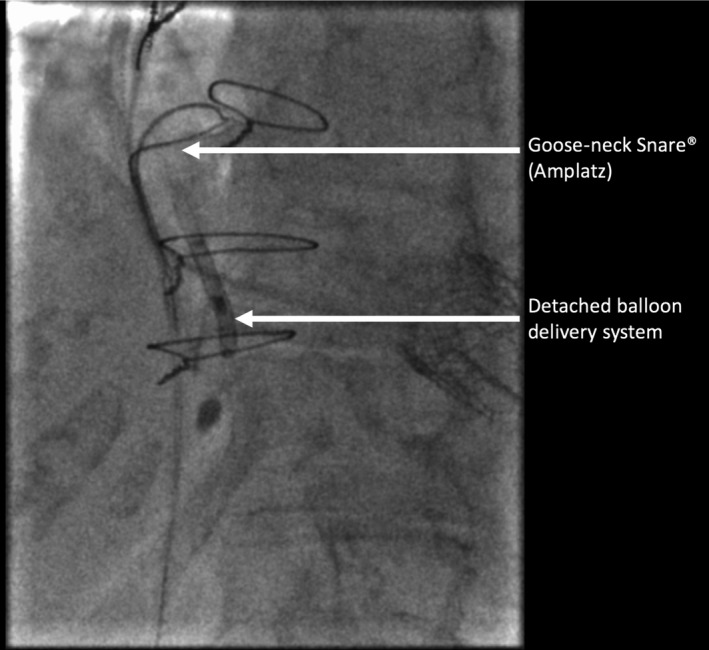
A Goose‐neck Snare^®^ (Amplatz) was able to securely grip the detached deployment apparatus. We were able to achieve this by changing to a femoral approach

**Figure 5:**
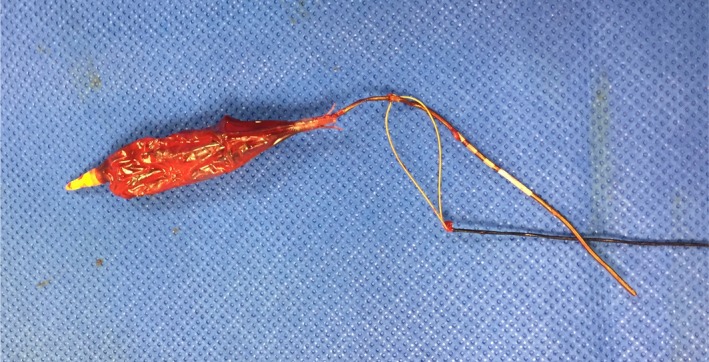
The externalized device can be seen attached to the Goose‐neck snare^®^. This was approximately 15 cm in length

We were unable to remove the balloon delivery system as it had become entangled in the left ventricular pacing lead. The cause was likely to have been the Edwards SAPIEN three heart valve having partially occluded the coronary sinus, resulting in entanglement of the left ventricular lead and balloon delivery system. We applied continuous traction via the RIJV, causing displacement of the left ventricular lead but the balloon delivery system remained wedged in place. We therefore cut the apparatus at the skin leaving it within the superior vena cava and right atrium (Figures 2 and 3). The options to remove the retained device were discussed, including surgical removal, and it was agreed to attempt percutaneous snaring of the device. Initially, we tried to snare the device from the RIJV with a Goose‐neck^®^ snare (Amplatz) but this was unsuccessful, so we proceeded to extract via the right femoral route. Given the length of the retained balloon delivery system, we selected a 30 mm Goose‐neck^®^ snare (Amplatz). Using fluoroscopic guidance, we were able to snare the apparatus and by applying gentle traction in different directions, this came away from the left ventricular lead. We were then able to remove it via the femoral vein in its entirety (Figure 4). Bleeding was controlled with a ProGlide^®^ device and manual pressure. The extracted device and balloon were approximately 15 cm in length (Figure 5). An echocardiogram, which was carried out the following day, demonstrated the TVIV was well seated with normal forward flow velocities and no significant TR. The patient made a good recovery after the procedure and was discharged home. Given the complications that had arisen, we decided to reposition the displaced left ventricular lead several weeks later. Unfortunately, we were unable to pass the stylet to the tip of the lead and, therefore, decided to abandon the old lead, and placed a new left ventricular lead in the high lateral vein. The patient made an uneventful recovery. She was reviewed in clinic several months following the intervention. Her symptoms had markedly improved, with less dyspnoea and peripheral edema.

## 3 DISCUSSION

The mainstay of treatment for severe TR is medical therapy, although in selected cases, surgical intervention can be beneficial.^5^ However, recurrence of TR following surgical intervention has been seen in 20% of patients at the 5‐year follow‐up appointment.^1^ Given the high surgical risk associated with redoing surgery, TVIV implantation for a dysfunctional BVR is becoming an increasingly appealing option. Transcatheter aortic implants have already been successfully placed in the aortic and mitral valve positions with good outcomes. As we become more experienced with the use of TVIV, this will improve procedural and patient outcomes.

This case describes the successful management of a complication arising from a TVIV procedure. The retained balloon delivery system was successfully removed percutaneously using snaring techniques and equipment, usually employed for percutaneous pacemaker/defibrillator lead extraction. This meant we could avoid open surgical extraction, which would have been associated with a significant risk of morbidity and mortality.^6^ To our knowledge, there are no reported cases involving intravascular detachment of the balloon delivery apparatus used to implant a TVIV. In this case, it was likely to have been caused by the pacing leads that prevented removal of the device from the RIJV. When we encountered this situation, we attempted a step‐wise extraction technique as advocated in the current HRS consensus document.^6^ Superior extraction using a Goose‐neck^®^ snare was not possible, and thus, we changed to a femoral route to enable us to use more traction and a larger snare. The balloon delivery apparatus was removed via this route, and the patient made a good recovery.

## 4 CONCLUSION

This case highlights the benefit to using percutaneous extraction techniques using femoral snaring. Given the rise in percutaneous valve implantations, this technique may have increasing importance in the future. As these cases can be complex, it emphasises the importance of experience in this setting when a significant complication occurs. To our knowledge, this is the first case report of a TVIV device detachment, treated by percutaneous extraction, which meant we could avoid surgical intervention.

## AUTHOR CONTRIBUTIONS

Dr Baldeep S. Sidhu: involved in concept/ design, drafted the article, and approval of submitted final version.Dr Justin Gould: involved in critical revision of article and approval of submitted final version.Dr Benjamin Sieniewicz: involved in critical revision of article and approval of submitted final version.Dr Bradley Porter: involved in critical revision of article and approval of submitted final version.Professor Bernard Prendergast: involved in critical revision of article and approval of submitted final version.Professor Simon Redwood: involved in critical revision of article and approval of submitted final version.Professor Christopher A. Rinaldi: involved in concept/ design, critical revision of article, and approval of submitted final version.
